# Revision Pulley Reconstruction in a Rock Climber for Phalanx Bone Resorption With Extensor Retinaculum Slip: A Case Report and Technique Review

**DOI:** 10.7759/cureus.71804

**Published:** 2024-10-18

**Authors:** Michele Cerasani, Hilary T Campbell, William Neal, William Wang, Jadie De Tolla

**Affiliations:** 1 Orthopedic Surgery, NYU Langone Orthopedic Hospital, New York, USA

**Keywords:** a2 pulley rupture, extensor retinaculum autograft, finger pulley reconstruction, palmaris longus autograft, revision reconstruction

## Abstract

We report this case of a rock climber who sustained a right ring-finger grade-III A2 pulley rupture. After failed nonoperative management, the patient underwent pulley reconstruction with ipsilateral palmaris longus autograft using a double-loop technique. The immediate postoperative course was uncomplicated, and the patient returned to painless rock climbing six months after the index procedure. One year postoperatively, the patient developed discomfort on the dorsum of the proximal phalanx which progressed to baseline pain at rest. Radiographs and MRI taken 16 months postoperatively demonstrated intact palmaris graft with underlying bone osteitis and resorption of the proximal phalanx. The range of motion remained full without bowstringing. The patient was indicated for revision A2 pulley reconstruction with ipsilateral extensor retinaculum autograft. Six months following revision pulley reconstruction, the patient was pain-free and radiographs demonstrated improvement in cortical density. The patient was able to return to rock climbing. Our case suggests pulley reconstruction with an extensor retinaculum slip sutured to the ever-present fibrous rim is a viable option with successful short-term outcomes in the setting of post-reconstructive bone resorption.

## Introduction

Closed traumatic ruptures of flexor tendon pulleys are rare in the general population, however, more commonly occur among rock climbers due to the unique biomechanical requirements for finger flexion while climbing. The flexor sheath pulley system permits normal flexor tendon function by forming a channel that keeps the tendon close to the bone to prevent bowstringing. When functioning properly, the pulleys turn linear translational force created by the muscle-tendon unit to torque at the joints, allowing for flexion at the interphalangeal joints. Disruption of one or more pulleys can result in bowstringing of the flexor tendon and thus loss of strength and digit range of motion [1]. A2 and A4 pulleys have traditionally been regarded as the most essential for preventing bowstringing [2], however, more recent biomechanical cadaver studies suggest the A2 and/or A4 pulleys may accept higher levels of partial disruption with less clinical deficiency than previously thought [3,4].

Typically, nonoperative treatment is trialed for most acute pulley ruptures, including grades I to III on the grading system for flexor pulley injuries proposed by Schoffl [5]. Surgical management is reserved for patients with grade IV injuries, single pulley injuries with associated lumbrical damage or collateral ligament rupture, and those with functional deficits secondary to pain or limited range of motion. When surgical management is indicated, the use of a palmaris longus graft or extensor retinaculum graft for pulley reconstruction has demonstrated promising outcomes [6].

Pulley ruptures that fail operative treatment represent a difficult clinical problem. Despite good outcomes in most cases of primary pulley reconstruction, some patients require revision pulley reconstruction due to persistent functional issues [7]. The complexity of the pulley and flexor tendon system in the setting of altered anatomy renders revision reconstruction a challenge. This case study represents a unique scenario of proximal phalanx bone resorption following reconstruction with a double-looped palmaris longus graft subsequently requiring revision pulley reconstruction with a slip of the extensor retinaculum.

## Case presentation

**Figure 1:**
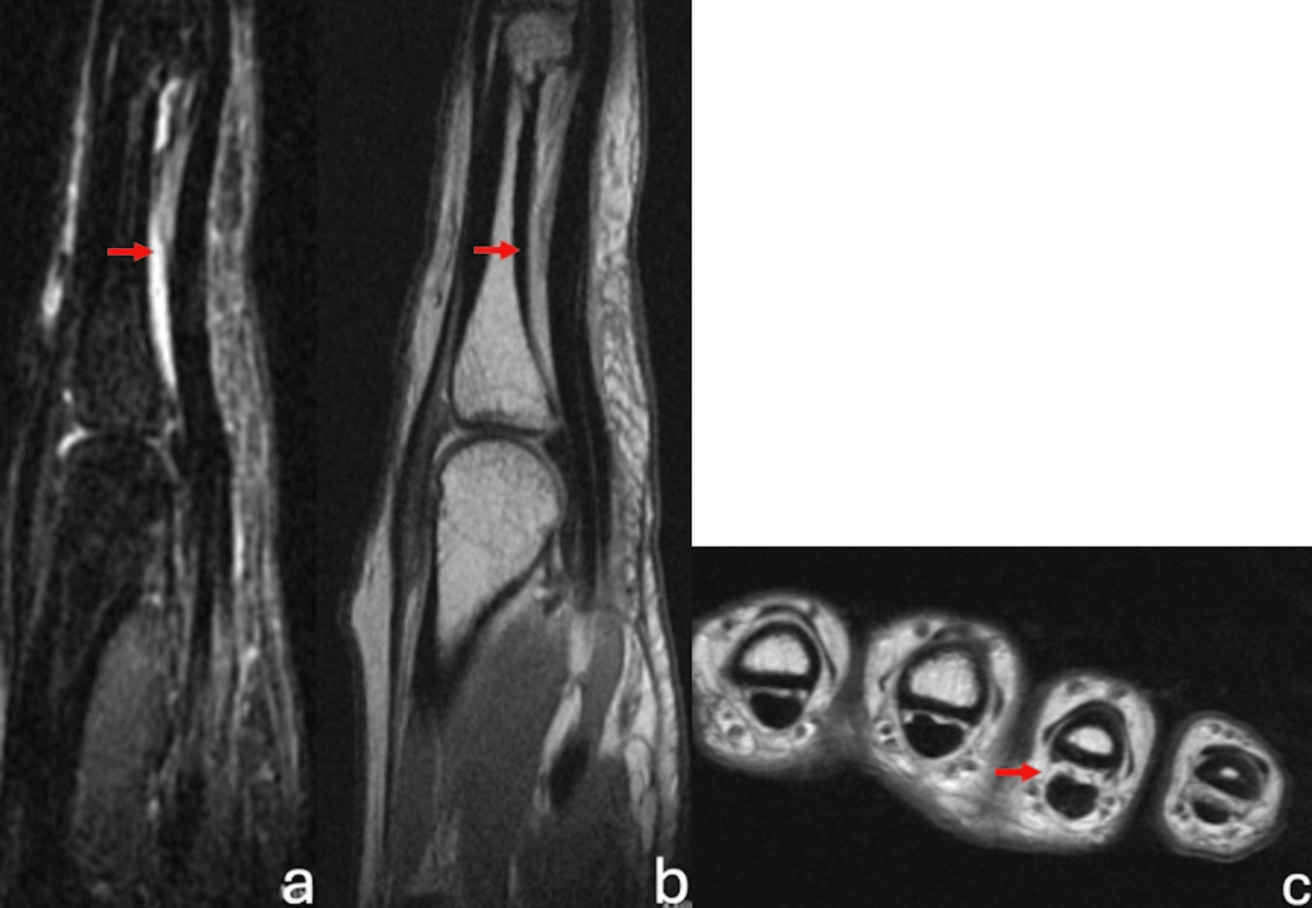
MRI following the initial injury demonstrating an A2 pulley rupture with increased tendon to phalanx distance indicated by the red arrows.

**Figure 2:**
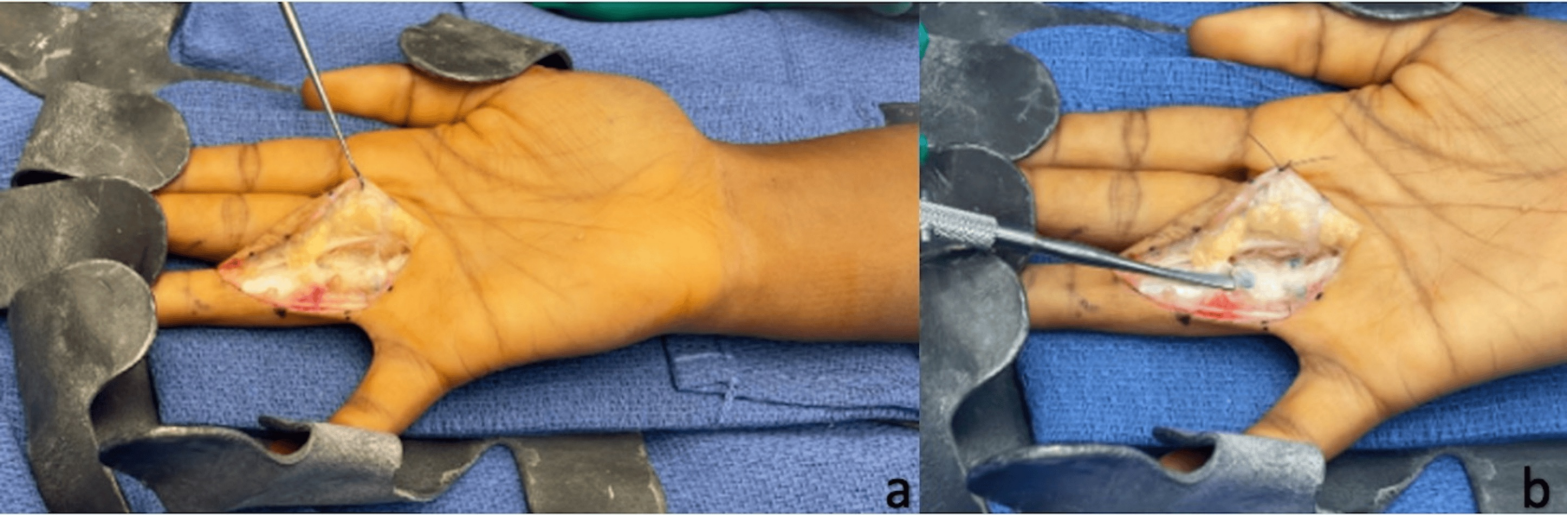
Pulley reconstruction with palmaris autograft.

**Figure 3:**
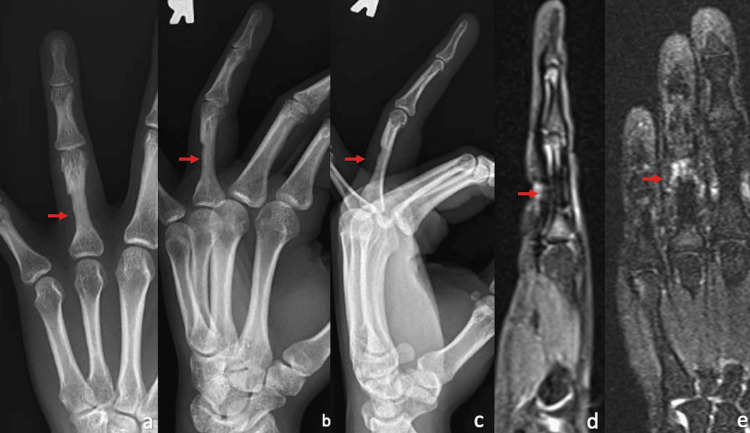
Radiographs (a-c) and MRI (d, e) demonstrating proximal phalanx bone resorption and osteitis (indicated by the red arrows) 16-months status post index pulley reconstruction with intact palmaris longus autograft.

The patient presented is a 30-year-old, female, right-hand-dominant rock climber who sustained a right ring-finger injury during a rock-climbing incident. She presented to an outside orthopedic hand surgeon and was diagnosed with a grade III (complete) A2 pulley rupture via ultrasound and MRI (Figure 1a-c) and was treated nonoperatively with a ring splint and cessation of climbing for three months. Due to continued pain, the patient underwent pulley reconstruction with ipsilateral palmaris longus autograft using a double-loop technique (Figure 2a-b). The immediate post-operative course and therapy were uncomplicated, and the patient returned to painless rock climbing six months after the index procedure. One year postoperatively, the patient developed discomfort on the dorsum of the proximal phalanx which progressed to baseline pain at rest. Radiographs and MRI taken 16 months post-operatively demonstrated intact palmaris graft with underlying bone osteitis and resorption of proximal phalanx (Figure 3a-e). The range of motion remained full without bowstringing. The patient was indicated for revision A2 pulley reconstruction with ipsilateral extensor retinaculum autograft.

**Figure 4:**
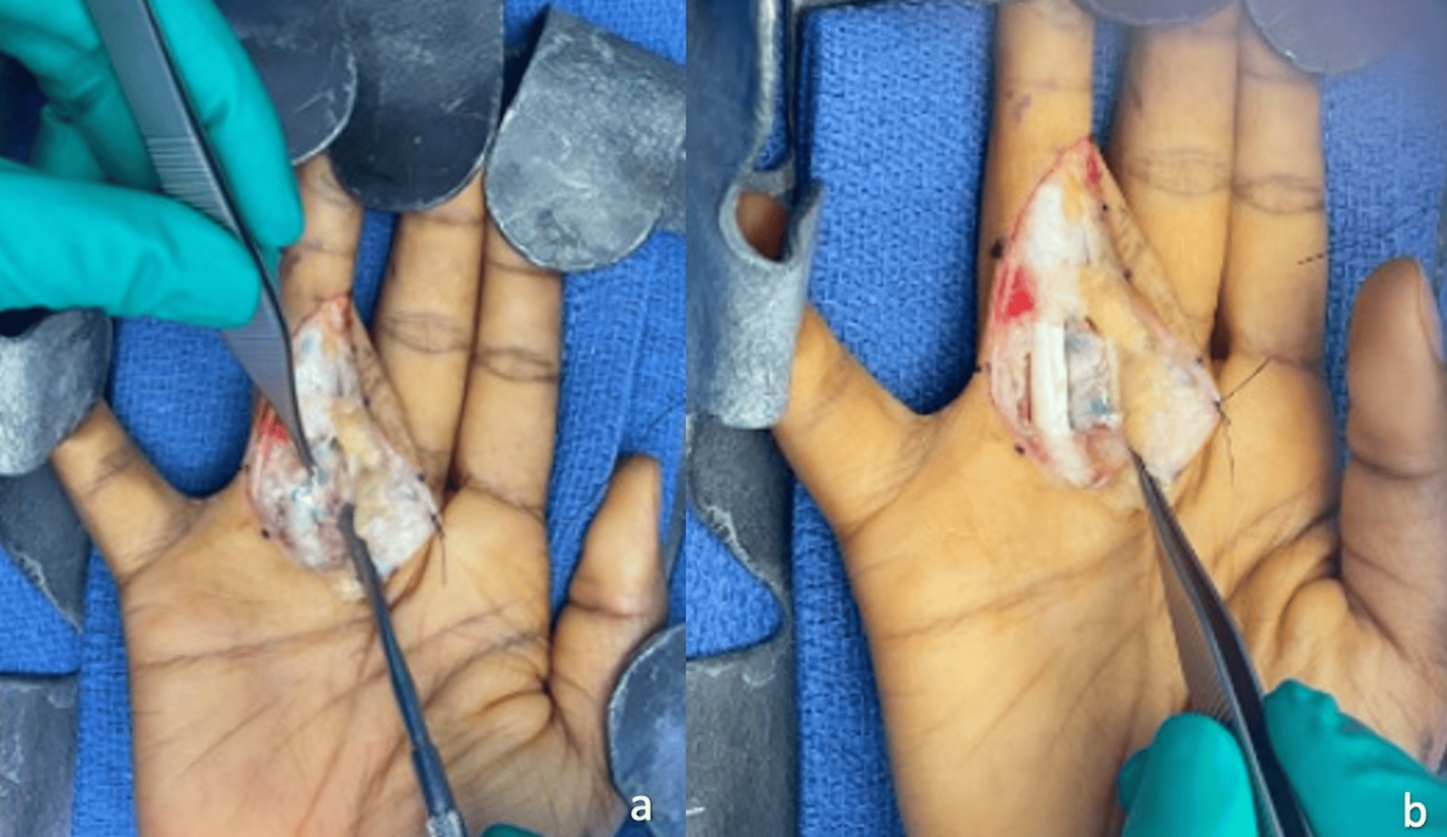
Palmaris graft excision and scar tissue debridement.

**Figure 5:**
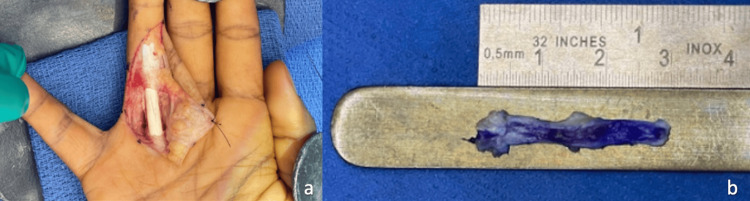
Ever-present fibrous rim identified and harvested slip of extensor retinaculum.

**Figure 6:**
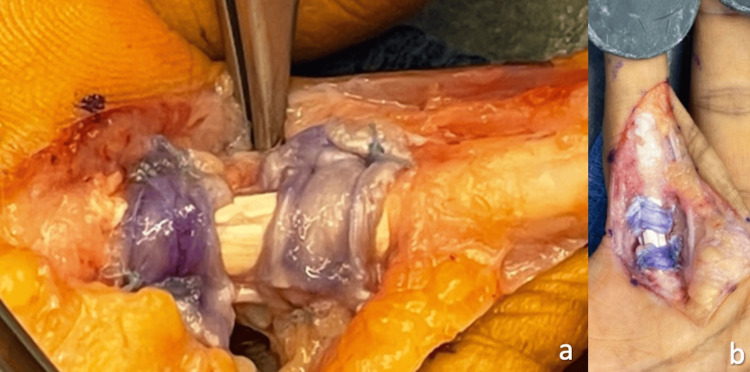
Extensor retinaculum sutured to ever-present fibrous rim.

The patient was indicated for pulley reconstruction given persistent functional limitations. The digital flexor sheath was approached utilizing the prior mid-axial incision and all scar tissue was adequately debrided. The A2 pulley with the intact palmaris double-looped autograft was identified. There were no restrictions on passive range of motion or tendon gliding. The palmaris graft was excised (Figure 4a-b). The ever-present fibrous rim was identified and then a 3-cm slip of ipsilateral extensor retinaculum was harvested (Figure 5a-b). The extensor retinaculum graft was then divided into two slips, each sutured to the fibrous rim of the proximal phalanx with six horizontal mattress sutures using 4-0 fiber wire (Figure 6a-b). Adequate tension was achieved by observing full, uninhibited passive flexion and extension of the digit without bowstringing. Enough space for a freer elevator beneath the revision autograft was left to ensure it was not over-tensioned. Postoperatively, the patient was placed in a dorsal blocking splint for two weeks and began therapy at that time with weight restrictions of two pounds or less.

**Figure 7:**
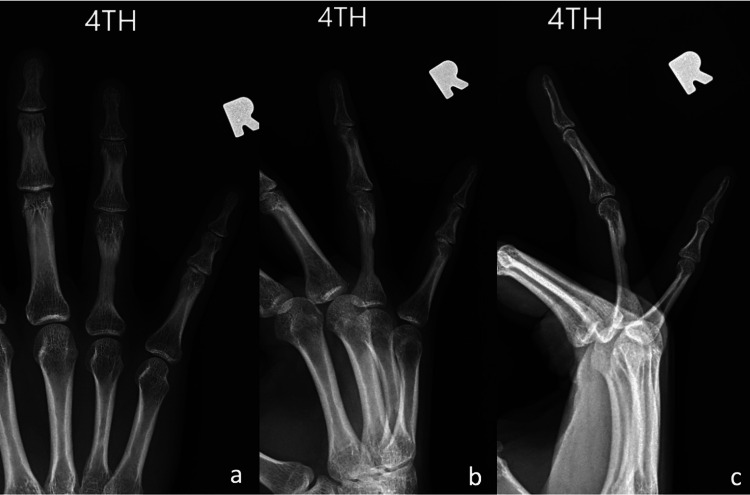
Radiographs six months postoperatively demonstrating improved cortical density of the proximal phalanx.

**Figure 8:**
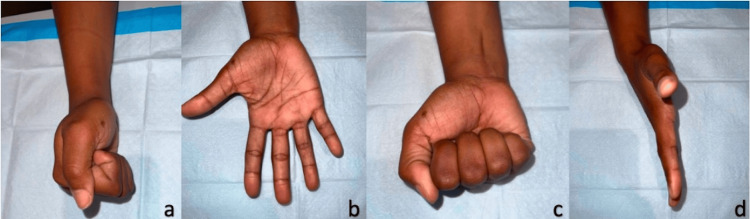
Full digit range of motion at 10 months postoperatively.

**Figure 9:**
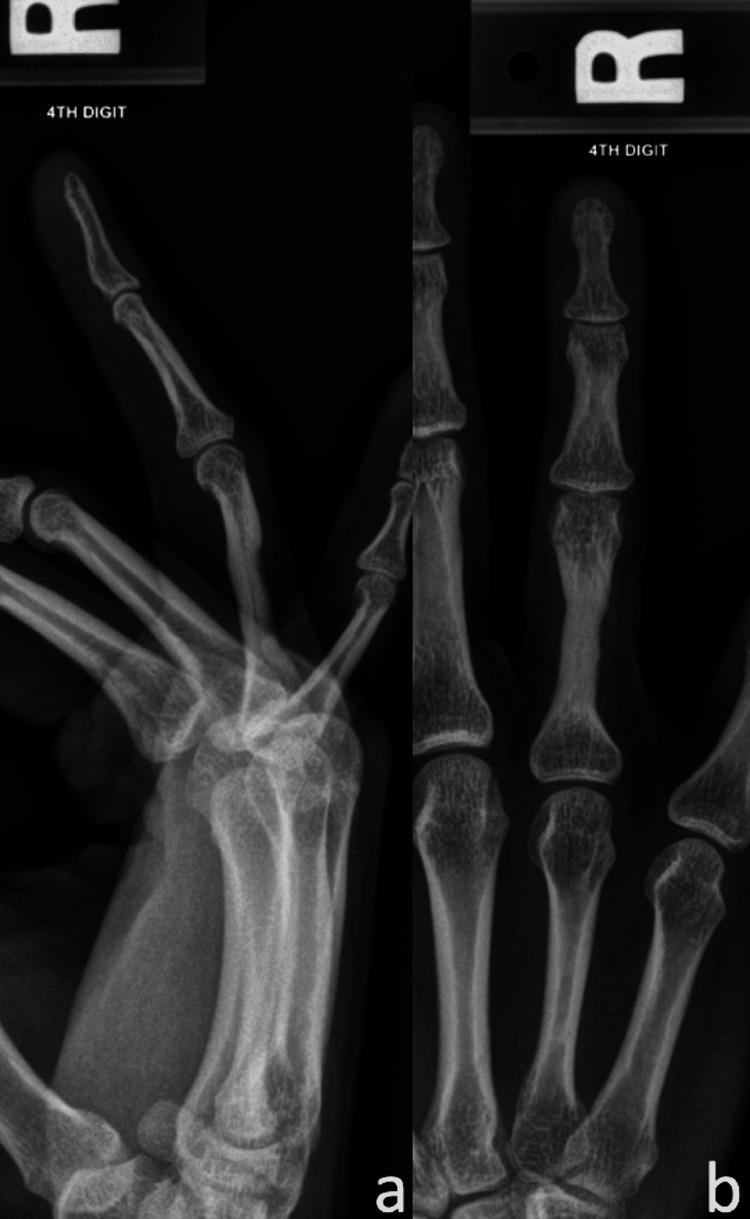
Radiographs at 10 months postoperatively demonstrating remodeling of the proximal phalanx with improved cortical density.

At two months, the patient's active range of motion was 0 to 90 degrees at the metacarpophalangeal joint (MCPJ), 15 to 90 degrees at the proximal interphalangeal joint (PIPJ), and 0 to 50 degrees at the distal interphalangeal joint (DIPJ). At three months, she had full digital range of motion and progressed to full weight bearing with therapy focused on grip strength - however, she still withheld from climbing. At six months, the patient was pain-free. Radiographs demonstrated improvement in cortical density (Figure 7a-c) and the patient was allowed to return to all activities including rock climbing with the understanding of increased fracture risk. At 10-month follow-up, she returned to pain-free rock climbing four to five times per week. On exam, she continued to have full digit range of motion (Figure 8a-d) and radiographs demonstrated remodeling of the proximal phalanx with improved cortical density compared to prior images (Figure 9a-b). 

## Discussion

This case presents a failed pulley reconstruction secondary to proximal phalanx resorption requiring revision with an extensor retinaculum autograft. There are a handful of case reports in the literature on proximal phalanx resorption following pulley reconstruction [8-10]. Lin GT describes a case of proximal phalanx resorption under a reconstructed A2 pulley using a 3-loop technique that did not remodel at 10 years follow-up [8]. One proposed mechanism of this complication is the shortened pulley and dissected periosteum resulting in impaired blood supply to the bone [9,11]. Additionally, Ferran and Dias discuss a report of proximal phalanx resorption in the digit that underwent pulley reconstruction using the loop method [10]. Currently, there is limited literature on proximal phalanx resorption and revision reconstruction using the extensor retinaculum. 

An extensor retinaculum graft was described by Gabl et al, where a 10 mm width strip of extensor retinaculum is harvested with a periosteal strip intended to aid in the biological healing of the phalanx [12]. The graft consists of collagen tissue and a synovial layer. They describe drilling bilateral burr holes of 1mm in diameter from the palmar aspect to the lateral side of the phalanx then placing the graft with the synovial layer inside creating a tight encircling of the flexor tendon. Subsequently, the graft is then fixed to the bone with a nonabsorbable suture [12]. The technique described in our case does not use drill holes but rather uses the fibrous rim of the index graft to anchor the new extensor retinaculum via horizontal mattress sutures. Considering the underlying proximal phalanx resorption, drilling into the diminished quantity and compromised quality of bone in the proximal phalanx presents a potential risk of intraoperative fracture and graft failure.

A crucial factor in both primary and revision pulley reconstruction involves graft choice. Arora et al compared extensor retinaculum graft and palmaris longus tendon in 23 patients with either isolated ruptures of the A2 pulley or combined A2 and A3 pulleys [6]. They found no statistical difference between the two groups regarding limitation in active range of motion, circumference of the finger, power, or pinch grip strength. There were no reported complications in their primary pulley reconstruction cohort [6]. We opted for the extensor retinaculum as our graft of choice, as numerous studies have shown successful outcomes when using this graft for an isolated pulley rupture [12-14]. Although these were primary cases, we demonstrate the successful use of extensor retinaculum for revision reconstruction. 

Furthermore, the choice of graft reconstruction technique holds significant importance. Oeckenpöhler et al. conducted a review of 23 patients who underwent pulley reconstruction using the Okutsu double- or three-loop technique, demonstrating good finger function in everyday life [7]. A biomechanical analysis of five different pulley reconstruction methods revealed that only the triple-loop reconstruction could withstand a load to failure comparable to that of a normal pulley [15].

Postoperative protocols are crucial for promoting healing. Schöffl et al. suggest an initial two-week immobilization period, followed by early functional motion while using a thermoplastic splint or a soft-cast ring for four weeks to safeguard the pulley [5]. Moreover, they advise the continuation of taping during sports activities for at least six months [5]. In our case, a comparable postoperative protocol was employed, with the patient placed in a dorsal blocking splint for four weeks, followed by an early range of motion.

## Conclusions

This case highlights the challenge of graft tensioning where an over-tensioned graft may disrupt the phalangeal blood supply leading to bone osteitis/resorption and potential fracture requiring revision surgery. Pulley reconstruction with an extensor retinaculum slip sutured to the ever-present fibrous rim is a viable option with successful short-term outcomes in the setting of post-reconstructive bone resorption. While nonoperative treatment with ring splints is highly successful in return to climbing for isolated pulley ruptures, there are several options regarding graft and technique choice when operative intervention is indicated.
